# Transcriptomics, Targeted Metabolomics and Gene Expression of Blackberry Leaves and Fruits Indicate Flavonoid Metabolic Flux from Leaf to Red Fruit

**DOI:** 10.3389/fpls.2017.00472

**Published:** 2017-04-06

**Authors:** Enrique Gutierrez, Ana García-Villaraco, José A. Lucas, Ana Gradillas, F. Javier Gutierrez-Mañero, Beatriz Ramos-Solano

**Affiliations:** ^1^Plant Physiology, Pharmaceutical and Health Sciences Department, Faculty of Pharmacy, Universidad San Pablo-CEU UniversitiesMadrid, Spain; ^2^Centre for Metabolomics and Bioanalysis, Pharmaceutical and Health Sciences Department, Faculty of Pharmacy, Universidad San Pablo-CEU UniversitiesMadrid, Spain

**Keywords:** *rubus*, secondary metabolism, flavonoids, flavonols, anthocyanins, translocation, transcriptomics

## Abstract

Blackberries (*Rubus* spp.) are among the high added value food products relevant for human health due to the increasing evidence of the beneficial effects of polyphenols, which are very abundant in these fruits. Interestingly, these compounds also play a role on plant physiology, being especially relevant their role in plant defense against biotic and abiotic stress. Hence, we hypothesize that since blackberry fruits have high amounts of flavonols and anthocyanins, leaves would also have high amounts of these compounds, and can be studied as a source of active molecules; furthermore, leaf synthesis would support their high contents in fruits. To explore this hypothesis, the present study reports a *de novo* transcriptome analysis on field grown blackberry leaves and fruits at the same time point, to establish the metabolic relationship of these compounds in both organs. Transcripts were aligned against *Fragaria vesca* genome, and genes were identified and annotated in different databases; tissue expression pattern showed 20,463 genes common to leaves and fruits, while 6,604 genes were significantly overexpressed only in fruits, while another 6,599 genes were significantly overexpressed in leaves, among which flavonol-anthocyanin transporter genes were present. Bioactives characterization indicated that total phenolics in leaves were three-fold, and flavonols were six-fold than in fruits, while concentration of anthocyanins was higher in fruits; HPLC-MS analysis indicated different composition in leaves and fruits, with cyanidin-3-glucoside as the only common compound identified. Next, RT-qPCR of the core genes in the flavonol anthocyanin pathway and regulatory MYB genes were carried out. Interestingly, genes in the flavonol-anthocyanin pathway and flavonol-transport families were overexpressed in leaves, consistent with the higher bioactive levels. On the other hand, transcription factors were overexpressed in fruits anticipating an active anthocyanin biosynthesis upon ripening. This suggests that, in addition to the biosynthesis taking place in the fruits during ripening, translocation of flavonols from leaves to fruits contributes to the high amounts of bioactives starting to accumulate in fruits.

## Introduction

Blackberry fruits are an important source of bioactive compounds, among which are flavonols, catechins, and anthocyanins (Garcia-Seco et al., 2013). These molecules have been reported to have beneficial effects to prevent metabolic syndrome at different levels such as cardiovascular disease and type II diabetes; hence, including blackberry on human diet is beneficial for health (Tavares et al., 2013; Feresin et al., 2016; Sarkar et al., 2016). On the other hand, they play a relevant role in plant defense against biotic and abiotic stress (Daayf et al., 2012). Catechins have also proved their improving endothelial function, hypertension, coronary heart disease, obesity, insulin resistance, as well as glucose and lipid metabolism (Kim, 2008).

Flavonols are a class of flavonoids, which have different roles in the green part of the plant, such as antioxidant, protecting the plant from reactive oxygen species (ROS), produced by abiotic stress like UV-B radiation, soil salinity, as a response to depletion of nitrogen in plants, as well as those ROS produced after biotic stress, as pathogen challenge, when they behave as phytoalexins (Wang and Lin, 2000; Gutiérrez-Mañero et al., 2015). They have also a growth regulating role, as they can act as auxin controllers, so they are able to control the individual plant organs and development of the whole plant (Agati et al., 2012; Falcone Ferreyra et al., 2012; Brunetti et al., 2013; Nakabayashi et al., 2014).

Despite the agricultural and biological importance of the genus *Rubus*, knowledge of their genetics and genome is very limited. *Rubus* spp. Var. Loch Ness is a high yielding tetraploid (4*n* = 28) blackberry, and one of the most widely cultivated varieties. However, and despite its high added economic value and as a source of bioactive compounds, its genome has not been sequenced yet. Therefore, other strategies need to be used to gain knowledge to improve production and health related benefits.

RNA-Seq has been recognized as a powerful tool to analyze the transcriptome of an organism that has not been completely sequenced (Wang et al., 2009). The sequencing of RNA has long been recognized as an efficient method for gene discovery and remains the gold standard for annotation of both coding and non-coding genes (Adams et al., 1991). Furthermore, the RNA-Seq method offers a holistic view of the transcriptome (Wang et al., 2010), and the information obtained from this wide analysis can be used to study specific pathways or physiological situations in a given species, in order to obtain useful information.

Many studies have addressed flavonoid synthesis, identification and subcellular accumulation mechanisms in different plant species as in grapevine (Perez-Diaz et al., 2016), soybean (Dastmalchi et al., 2017), banana (Dong et al., 2016) among others (Zhao, 2015) resulting in characterization of many specific transporters in different organs and accurate flavonoid profiles. Long distance transport has been demonstrated in *A. thaliana* (Buer et al., 2007) And the possibility of long distance flavonoid translocation in grapevine has been suggested, based on the presence of a specific transporter on the vascular bundles, which expression increases from veraison to harvest (Petrussa et al., 2013). Most studies on blackberry and other berries (Paredes-López et al., 2010) have been done on fruits since they are the edible part and therefore, hold the health-potential. The biosynthesis of flavonols and anthocyanins in blackberry fruits along maturation has already been described, demonstrating an increased biosynthesis of anthocyanins in the black ripen stage (Garcia-Seco et al., 2015). On the other hand, it may be anticipated that blackberry leaves also have an active secondary metabolism, in which flavonols must be abundant to participate in adaptation to environmental changes.

Based on this background, the aim of this study is focused on (i) the relevance of the transcriptome to envisage the specific situation of leaves and red fruits at the same time point, and (ii) to establish the relationship of the flavonol-anthocyanin pathway core and regulatory gene expression and their contents in two different organs, given the relevance of these metabolites for defense in leaves and for human health in fruits. In this work, we describe the transcriptome of leaves and red fruits by alignment to *Fragaria* as a reference genome sequence from the same family (*Rosaceae*); then, both transcriptomes are compared, quantification of core and regulatory genes of the flavonol-anthocyanin pathway expression by RT-qPCR, and HPLC-MS analysis of leaves and fruits is carried out. Differential gene expression suggests that leaf-flavonols are translocated to fruits during the early stages of maturation to achieve the high amounts accumulated therein on ripe fruits.

## Materials and methods

### Plant materials, growth conditions, and RNA extraction

The *Rubus* spp. cv. Loch Ness plants used in this study were kindly provided by Agricola El Bosque S.L. “La Canastita” (Lucena del Puerto, Huelva, Spain). Plants and greenhouses were handled according to regular agricultural practices (Ramos-Solano et al., 2014). Plants were grown in Huelva (South Eastern Spain) from September 2014 to February 2015 under “winter cycle.” Shortly, plants undergo an artificial cold period before transplant to greenhouses in order to start their regular cycle. Blackberry cycle has three stages: vegetative, flowering and flowering-fruiting; the duration of these stages is variable depending on the transplant moment, and each average one third of the plant's life. In this experiment, plants were transplanted at the end of September, flowering in November and fruiting in January when flowers, green, red and black fruits are present in the plants at the same time. Fruits were sampled in red stage. Leaves and fruits were immediately frozen with liquid nitrogen and brought to the lab. Three replicates were taken, being each one constituted from plant material of 20 plants; a total of 25 leaves and 125 g of ripening fruits were randomly sampled and pooled constituting the replicates.

Prior to RNA extraction, samples were removed from the −80°C freezer and grounded to a fine powder with liquid nitrogen using a sterilized mortar and pestle. Total RNA was isolated from each replicate (red fruits and leaves) with Plant/Fungi Total RNA Purification kit (50) (NORGEN™) (DNase treatment included) and after confirmation of RNA integrity using Nanodrop™ and Experion™, total RNA was sent to Exiqon™ for sequencing.

### RNA-Seq

#### Quality control and library preparation

RNA obtained from the three biological replicates were pooled and separated in three analytical replicates for RNA-Seq analysis. RNA samples were DNase treated and extracted as described before. Thirty microliters of RNA samples were passed through quality control with Nanodrop™ and Experion™, after that total RNA meeting quality criteria was sent to Exiqon™ for sequencing. A total of six libraries were done, three form leaves and three from fruits.

During the library preparation, poly-A tailed transcripts were enriched, as mRNA sequencing targets this type of transcripts, using an Oligo-dT magnetic bead-based system were enriched. The poly-A tailed transcripts include the coding mRNAs (1–4% of the whole transcriptome), so by this enrichment the appropriate depth of the sequencing for coding mRNA was achieved. The library preparation also retains information of which of the two strands of DNA was used to transcribe the given RNA, which enables the detection of antisense transcript expression. Mitochondrial poly-A tailed transcripts were bioinformatically filtered since they were considered to be high abundance sequences. The sequencing was paired end, which increases the mapping percentage to poorly annotated genomes, and identifies splice variants.

Two types of sequencing library quality controls were performed, firstly after the library preparation and bead based size selection, the size distribution of the library was evaluated using a Bioanalyzer high sensitivity DNA chip. Then, qPCR based quantification of each library was performed, and samples were normalized and pooled in equimolar ratios. After pooling of sample libraries, qPCR based quantification was performed on the library pool to ensure optimal concentration for cluster generation on the flow cell.

Mapping of the sequencing data represents a useful quality control step in the Next generation sequencing (NGS) data analysis pipeline as it can help to evaluate the quality of the samples. For this purpose, reads were classified in the following classes (i) outmapped reads or high abundance reads: rRNA, mtRNA, polyA, and PolyC homopolymers; (ii) unmapped reads: no alignment to reference genome possible; (iii) mappable reads: alignment to reference genome possible. In a typical experiment it is possible to align 60–90% of the reads to the reference genome. However, this number depends upon the quality of the sample and the coverage of the relevant reference genome; if the sample is degraded, fewer reads will be mRNA specific and more material will be degraded rRNA. In this case where the reference genome is different from the host organism, the mapping fraction is expected to be significant lower. To test the differences between the blackberry and strawberry, we have taken reads that align to blackberry contig sequences, these reads should thus be *bona fide* blackberry sequences, and aligned them to the strawberry genome. Following this procedure, 30% of the reads are expected to be mapped to strawberry genome with the relatively loose stringency settings.

#### Sequencing

The library pools to be sequenced were denatured and diluted/neutralized in the required concentrations. Then, cluster generation was performed on the appropriate flow cell using single molecule clonal amplification. Finally, the high- throughput NGS was performed using the Illumina sequencing technology platform.

#### Transcriptome aligned

The raw data (cDNA) was aligned to the reference sequence, using as reference the sequence of wild strawberry (*Fragaria vesca* subesp. *Vesca*) a species of same family (*Rosaceae*), but from a different genus, *Fragaria*.

The strawberry genome and gene information were downloaded from the Washington State University and Clemson University website (http://www.rosaceae.org), funded by the 2009 USDA NIFA Specialty Crop Research Initiative Program.

#### Annotation and classification of genes

Raw data (75 bp reads) was generated by Exiqon™ and used for the annotation, aligning the reads against the reference genome of the Strawberry, *Fragaria vesca* Whole Genome v2.0.a1 Assembly (Annotation reference: *Fragaria vesca* Whole Genome v2.0.a1 Assembly & Annotation; The strawberry genome and annotation was found at: https://www.rosaceae.org/species/fragaria_vesca/genome_v2.0.a1). By this method the identification of the genes was achieved.

Genes were then used for BLAST searches and annotation against NCBI Nr protein database (NCBI non-redundant sequence database). Blast was performed by CloudBlast, using Blastx-fast Blast Program, Non-redundant protein sequences (nr) from 17.01.2016 as Blast DB (Blast database), with a blast expectation value of 1 × 10^−10^, a word size of 6, and a HSP length cutoff of 33. Gene sequences were further aligned by BLASTX to protein databases such as Swiss-Prot, and KEGG, retrieving proteins with the highest sequence similarity with the given genes along with the functional annotations for their proteins. If results of different databases conflicted, a priority order of Nr, Swiss-Prot, and KEGG was followed. For everything previously described, Blas2GO® was used (Conesa and Gotz, 2008). The Blas2GO® program was also used to obtain GO annotations for the genes. The Blast2GO software was then used to perform mapping and annotation (mapping is used to look for associated GO terms to the Hit Blast, and annotation selected from this GOs those with good statistical support). Annotation was made with an annotation cutoff of 55, and a GO weight of 5.

#### Expression levels

Gene expression levels were calculated using Fragments Per Kilobase of transcript per million Mapped reads (FPKM) method. The formula used is FPKM= 10^9^^*^ C/(N ^*^ L), where C is the number of mappable reads that fell onto the gene's exons, N the total number of mappable reads in the experiment and L the number of base pairs in the exon. The log_2_ (fold-change) is the log-ratio of a gene's or a transcript's expression values in two different conditions. The log_2_ fold change is used to confirm the significance of the differential expression between the different samples [log_2_ (fold change) ≥ 1 (overexpression in fruit), or log_2_ (fold change) ≤ −1 (overexpression in leaves)].

### RT-qPCR analysis of flavonols and anthocyanins

In order to confirm the expression obtained in the RNA-Seq data, a RT-qPCR was performed. The retrotranscription was performed using iScript tm cDNA Synthesis Kit (Bio-Rad). All retrotranscriptions were performed using an GeneAmp PCR System 2700 (Applied Biosystems): 5 min 25°C, 30 min 42°C, 5 min 85°C, and hold at 4°C. The amplification were performed with a MiniOpticon Real Time PCR System (Bio-Rad): 3 min at 95°C and then 39 cycles consisting of 15 s at 95°C, 30 s at 50°C and 30 s at 72°C, followed by melting curve to check the results. To describe the expression obtained in the analysis, cycle threshold (Ct) was used. Standard curves were calculated for each gene, and the efficiency values ranged between 90 and 110%. Two reference genes were used, actin for fruits and histone for leaves. A different reference gene was used for each organ, since no common gene was found meeting criteria for a reference gene. However, these genes have similar Ct, so they can be compared. Core and regulatory genes studied and the primers used for each appear in Table 1.

**Table 1:** **Primers designed to RT-qPCR expression analysis (Garcia-Seco et al., 2015)**.

**Gene**	**Forward primer**	**Reverse primer**
*Ru4CL*	5′CCAGAAGTTCGAGATCAACAAGT	5′GTCGGGACACTTGGTAATAGACA
*RuACT*	5′ATGTTCCCTGGTATTGCAGAC	5′CCACAACCTTGATCTTCATGC
*RuANR*	5′TCGCAATGTACTTCCAAGAAAC	5′CTTCATCAGCTTACGGAAATCAC
*RuANS*	5′TTGGTCTGGGATTAGAAGAAAGG	5′CTGAGGGCATTTTGGGTAGTAAT
*RuC4H*	5′CATCTGTAGGGAAGTGAAGGAGA	5′ACTTCAACCCTTCGTTAGTTGTG
*RuCHI1*	5′CAAGAAGGATTCCATCATCACA	5′CTCCACTTTGATCTTTGACGACT
*RuCHI2*	5′GAGGCAGTTCTTGAGTCAATCAT	5′CACGCTATCATCACTCACTTTCA
*RuCHS*	5′ATGGTGGTTGTTGAAATTCC	5′CTGGATTGCACACCCAGGTGGCCC
*RuDFR*	5′AATCAGAAGAAGGTGAAGC	5′CATTAKSACAAGTTTGGTG
*RuF3′5′H*	5′ATGCCHCATGYYDCCTTAGCHAAAATGG	5′TGGGCAATHGGRMGAGAYCC
*RuF3H*	5′ATGGCTCCTACACCTACTAC	5′TGGATCACCGTTCAACCTGTGGAAGG
*RuF3′H*	5′CCTATCTCCAAGCTGTCATCAAG	5′GTGGTATCCGTTGATTTCACAAC
*RuFLS*	5′CCTACAGGGAAGTCAATGAGAAA	5′CACATGGGATTTCAGTACCTTCT
*RuGST1*	5′TACTAGAATCACAAGCACCAGCA	5′ACCCAAAACTCACATAGACAACG
*RuGST2*	5′GAACTCATTGCTTGAGAGCGTAG	5′GATCTTCCACACTTCCTCTACCA
*RuLAR*	5′GTGGAGTCCCATACACGTACATT	5′CTGAAACTGATCTAACGGTGGAA
*RuPAL1*	5′GAGGAACTGGGGACTAGTTTGTT	5′AGCAGAGGATCAATCAGCTTTC
*RuPAL2*	5′GACTTGCTCTTGTTAATGGCACT	5′GAAAATCGCAGACAAGATTTCG
*RuMYB1*	5′CTCATTGACAGGAACAGGTGTC	5′CCTACAACAACACCAACGAGAAT
*RuMYB3*	5′GAGCTGTAGGTTAAGATGGACAAA	5′GTTTCCAAGGATAGAATGGAGATG
*RuMYB4*	5′ACAGCTCAGGACTCTGCTACAAC	5′GGTTTATAGACTCTTTGCCCACA
*RuMYB5*	5′ACTCAATCCAGACTCCTCATCTG	5′AGGAAGTGATTGGACTTTTAGGG
*RuMYB6*	5′TCCTATGGAGTACTTCCAAGCTC	5′TATGGCTGTTTAGTCCTCCTTGA
*RuTTG1*	5′GTTGTGATCTTGGATATCCGTTC	5′GAACAAATGTGCCTATAACTCTGC

To evaluate RT-qPCR values, one-way ANOVA was performed to compare core gene expression and transcription factors in the different organs, using Statgraphics plus 5.1 for Windows. Prior to ANOVA analysis homoscedasticity and normality of the variance was checked with Statgraphics plus 5.1 for Windows, meeting requirements for analysis.

### Phenolics and flavonoids characterization

#### HPLC-MS phenolics and flavonoids characterization

##### Standards and solvents

Phenolic acids including, gallic acid, caffeic acid, ferulic acid, and chlorogenic acid were purchased from Sigma (St. Louis, MO, USA) and flavonoids including, kaempferol, kempherol-3-*O*-rutinoside, kempherol-3-*O*-glucoside, quercetin, quercetin-3-*O*-rutinoside, quercetin-3-*O*-glucoside, (+)-catechin, (−)-epicatechin and cyanidin-3-*O*-glucoside, were purchased from Sigma and from Extrasynthese Co. ™ (Geney, France).

The standard solutions (10 ppm) were prepared in methanol. All the solvents, as methanol and acetonitrile (*Honeywell Riedel-de Haen*), were LC-MS grade. Purified water was obtained from Milli-Q Plus™ System from Millipore (Milford, MA, USA). Formic acid was purchased from Aldrich (St. Louis, MO, USA).

##### Sample preparation

The extraction of phenolics was performed as follows: 300 mg of powder were added to 300 μL of methanol. The mixture was vortexed for 2 min, sonicated for 5 min and centrifuged at 3,500 rpm for 5 min at 4°C. The supernatants were then collected and stored at −20°C until use for LC/MS analysis, and then the extracts were diluted in methanol solution at a ratio of 1:10 (v/v).

##### LC/MS analysis

Phenolic acids and flavonoids were analyzed by LC-MS-IT analysis. The identification of compounds was performed using an Agilent *1100 HPLC* system (*Agilent Technologies*) connected to a *Bruker Daltonics* esquire 3,000^plus^ *ion trap mass spectrometer* (*Bruker Daltonics*) with an electrospray interfase (ESI).

Separation was on a Kinetex XB-C18 column (100 × 2.1 mm, 2.6 μ; Phenomenex) running the following gradient of acetonitrile (solvent B) vs. 0.1% aqueous formic acid (solvent A): from 0.0 to 1.0 min 2% B, from 1.0 to 6.0 min 10% B, from 6.0 to 26.0 min 30% B, from 26.0 to 36.5 min 90% B, from 36.5 to 37.5 90%B, from 37.5 to 38.0 min 2% B, and from 38.0 to 45.0 min 2% B, with a flow rate at 0.3 mL/min and column temperature at 60°C Peaks were detected by UV/visible light absorbance, collecting chromatograms at 260 nm.

The data acquisition software employed was Esquire Control 5.2. Data analysis was performed using the Data Analysis 3.1 software (Bruker Daltonics) and were adquired in negative and positive ionization mode with the following experimental conditions: nitrogen gas temperature: 350°C; drying gas flow rate: 11.5 L/min and capillary voltage: ±4000 V; nebulizing pressure: 25 psi. Mass spectra were recorded using the full scan mode in the range of m/z 50–1,300.

#### Total phenols

Total phenols were quantitatively determined with Folin-Ciocalteu agent (Sigma. Aldrich, St Louis, MO) by a colorimetric method described by Xu and Chang (2007) with some modifications, gallic acid was used as standard (Sigma-Aldrich, St Louis, MO). One milliliter of extract was mixed with 0.250 mL of Folin-Ciocalteu 2N (Sigma. Aldrich, St Louis, MO) and 0.75 mL of Na_2_CO_3_ 20% solution. After 30 min at room temperature, absorbance was measured at 760 nm with an UV-Visible spectrophotometer (Biomate 5). A gallic acid calibration curve was made (*r* = 0.99). Results are expressed in mg of gallic acid equivalents per 100 g of fresh weigh (FW). All samples were measured in triplicate.

#### Total flavonols

Total flavonols were quantitatively determined through the test described by Jia et al. (1999), using catechin as standard (Sigma-Aldrich, St Louis, MO). One milliliter of extract was added to a flask of 10 mL with 4 mL of distilled water. After that 0.3 mL of NaNO_2_ 5%, and 0.3 mL of AlCl_3_ 10% were added after 5 min. One minute later, 2 mL of NaOH 1 M were added, and distilled water was added till 10 mL of total volume. The solution was mixed and measured at 510 nm with an UV-Visible spectrophotometer (Biomate 5). A catechin calibration curve was made (*r* = 0.99). Results are expressed as mg of catechin equivalents per 100 g of fresh weigh (FW). All samples were measured in triplicate.

#### Total anthocyanins

Total anthocyanins were quantitatively determined through the pH differential method described by Giusti and Wrolstad (2001). Extracts were diluted in pH 1 buffer (0.2 M KCl) and pH 4.5 (1M CH_3_COONa) in 1:15 proportion. After that, absorbance was measured at 520 and 720 nm respectively, in a UV-Visible spectrophotometer (Biomate 5). A cyanidin-3-glucoside calibration curve was made (*r* = 0.99). Results are expressed in cyanidin-3-glucoside equivalents per 100 g of fresh weigh (FW). All samples were measured in triplicate.

## Results

### Transcriptome sequencing and assembly

To construct a *de novo* transcriptome database, six mRNA libraries were generated by Illumina sequencing, three from leaves and three from red fruits of *Rubus* spp. cv. Loch Ness.

Table 2 summarizes mapping results. In addition to these, it also shows the total number of reads obtained for each sample. On average, 57 million reads (75 bp read length) were obtained from each sample and genome mapping was on average 26% for all samples. The uniformity of the sample's mapping indicates that samples are comparable.

**Table 2:** **Total reads of the three different replicates from fruit and leaves**.

**Sample name**	**Total readcount**	**rRNA (%)**	**Other (mtRNA)**	**Mapped (%)**	**Unmapped (%)**
Fruit 1	65264074	0.001	0.019	25.399	74.598
Fruit 2	52300955	0.001	0.017	25.499	74.498
Fruit 3	42196474	0	0.014	25.199	74.798
Leaves 1	43184377	0	0.138	26.374	73.528
Leaves 2	43979951	0	0.174	25.671	74.216
Leaves 3	49606567	0.001	0.303	27.359	72.492

*Total Readcount, %rRNA, % other RNA, % Mapped RNA, and % Unmapped RNA*.

**Figure 1:**
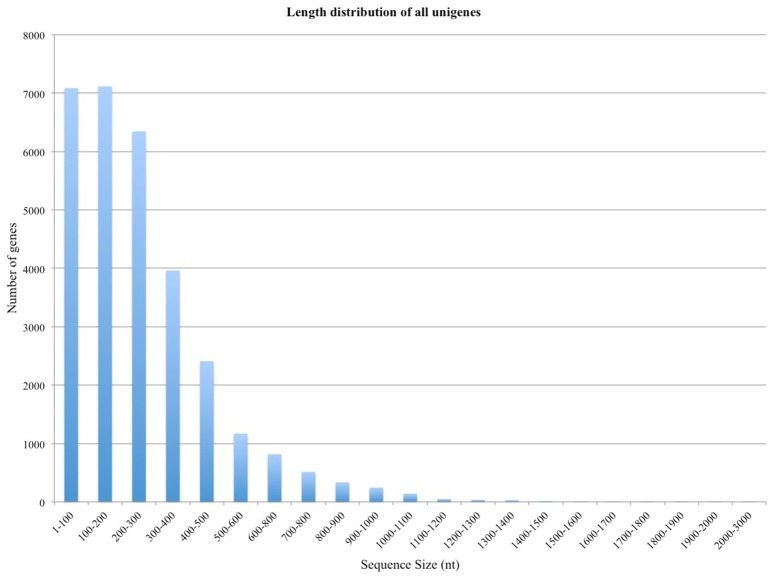
**Length distribution of the unigene assembly in the ***Rubus*** leaves and fruits transcriptome**.

In a typical experiment it is possible to align 60–90% of the reads to the reference genome, however, this number depends upon the quality of the sample and the coverage of the relevant reference genome; if the sample is degraded, fewer reads will be mRNA specific and more material will be degraded rRNA. In this case, in which the reference genome is different from the host organism, the mapping fraction was expected to be significantly lower. We found that 26% of the reads could be mapped to strawberry genome with relatively loose stringency settings. Mapping Alignment of paired-end reads to the genome was carried out against the *Fragaria vesca* Whole Genome v2.0.a1. The strawberry genome and annotation was found at: https://www.rosaceae.org/species/fragaria_vesca/genome_v2.0.a1. A total of 285 genes were >1,000 bp in length, and 30,001 were <1,000 bp (Figure 1).

**Figure 2:**
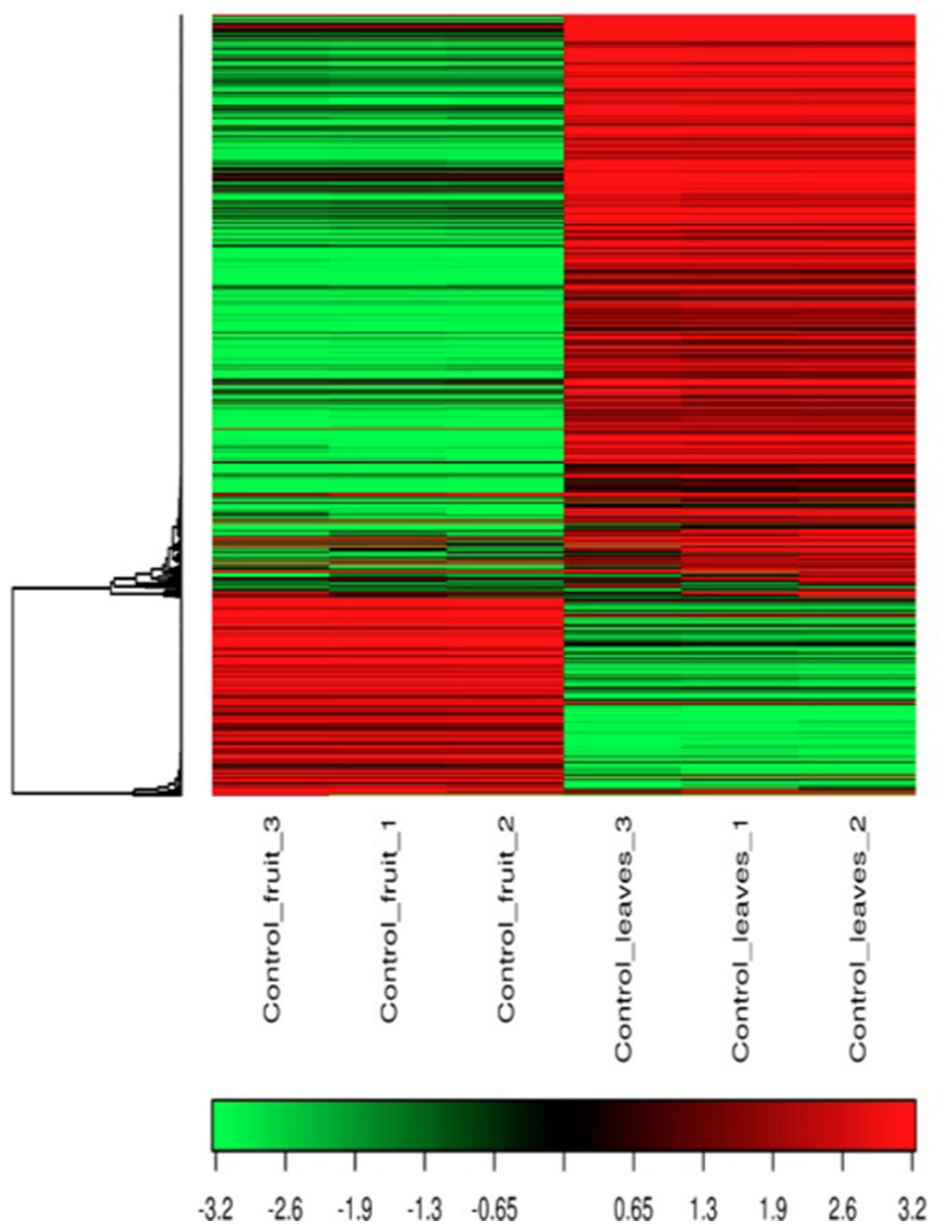
**Heat Map and unsupervised hierarchical clustering by sample; top 500 genes that have the largest coefficient of variation based on FPKM counts**.

**Figure 3:**
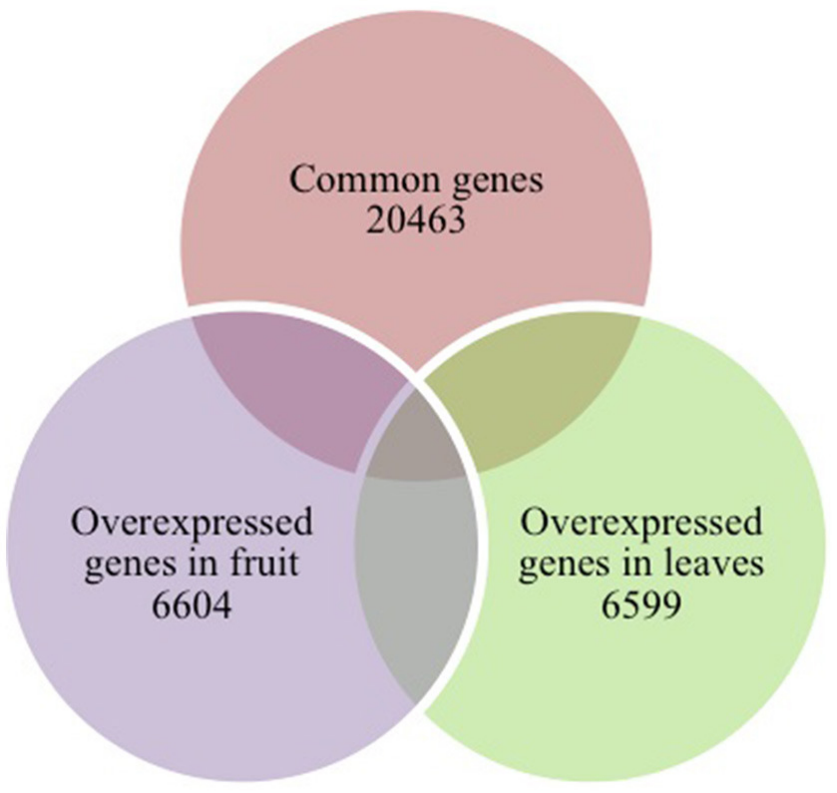
**Venn diagram of tissue-specific overexpressed and common genes**.

After sequencing and mapping alignment, normalized expression and differential expression (fruit vs. leaves) identified a total of 33,666 genes. The heatmap diagram (Figure 2) shows the result of the two-way hierarchical clustering of RNA transcripts and samples. It includes the 500 genes that have the largest coefficient of variation based on FPKM counts. Each row represents one gene and each column represents one sample. The color represents the relative expression level of a transcript across all samples. The color scale is shown below: red represents an expression level above the mean; green represents an expression level below the mean. When tissues were compared, the expression pattern showed that 20,463 genes were common to both organs (expression without significant differences), and 6,604 genes were significantly overexpressed in fruit, while 6,599 genes were significantly overexpressed in leaves (Figure 3).

### Blast, mapping, annotation, and interproscan

**Figure 4:**
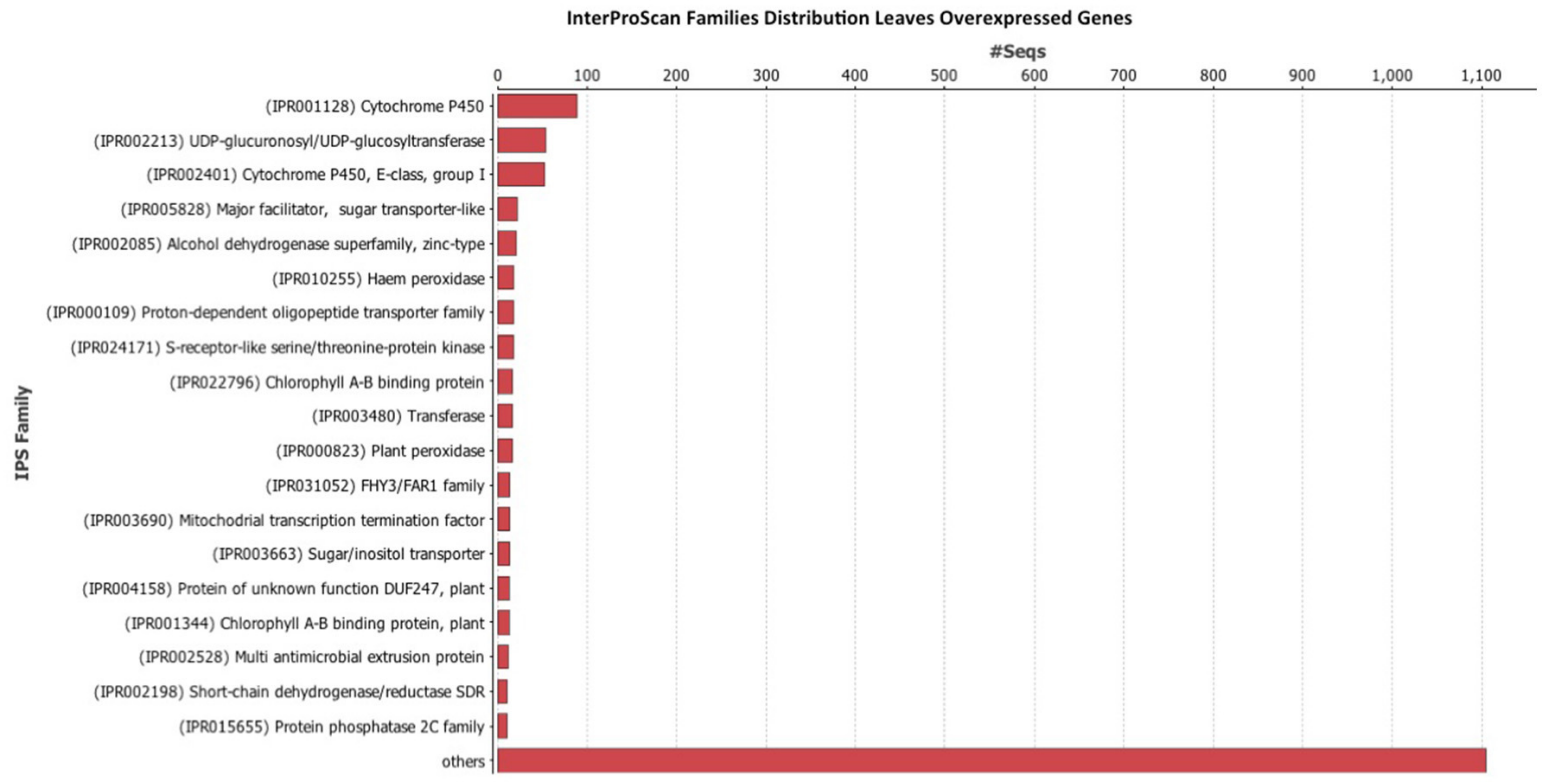
**Histogram of InterProScan analysis of ***Rubus*** spp. genes in leaves**.

**Figure 5:**
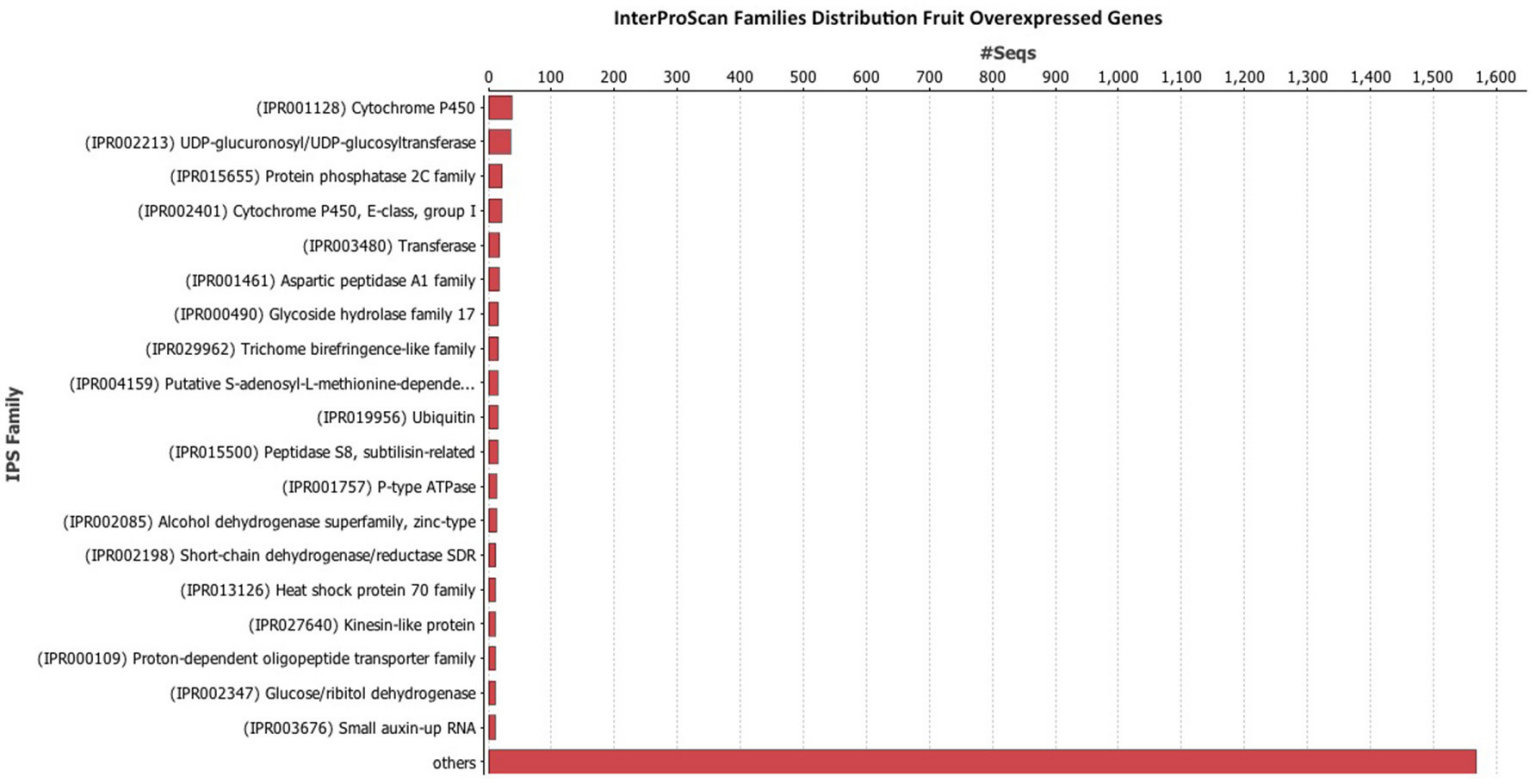
**Histogram of InterProScan analysis of ***Rubus*** spp. genes in fruit**.

Once the data was filtered, Blast2Go was used to perform the blast, mapping, annotation, and InterProScan. The InterProScan analysis gives a functional analysis of proteins, classifying them in families, and predicting important sites and domains. InterProScan analysis indicates that the Cytochrome P450 family is very abundant in leaves (Figure 4) and fruits (Figure 5). Also relevant the presence of glycosiltransferases, that appear in second position in both organs, and a general sugar transporter in leaves in fourth position. InterProScan analysis of common genes is shown in supplementary material.

**Figure 6:**
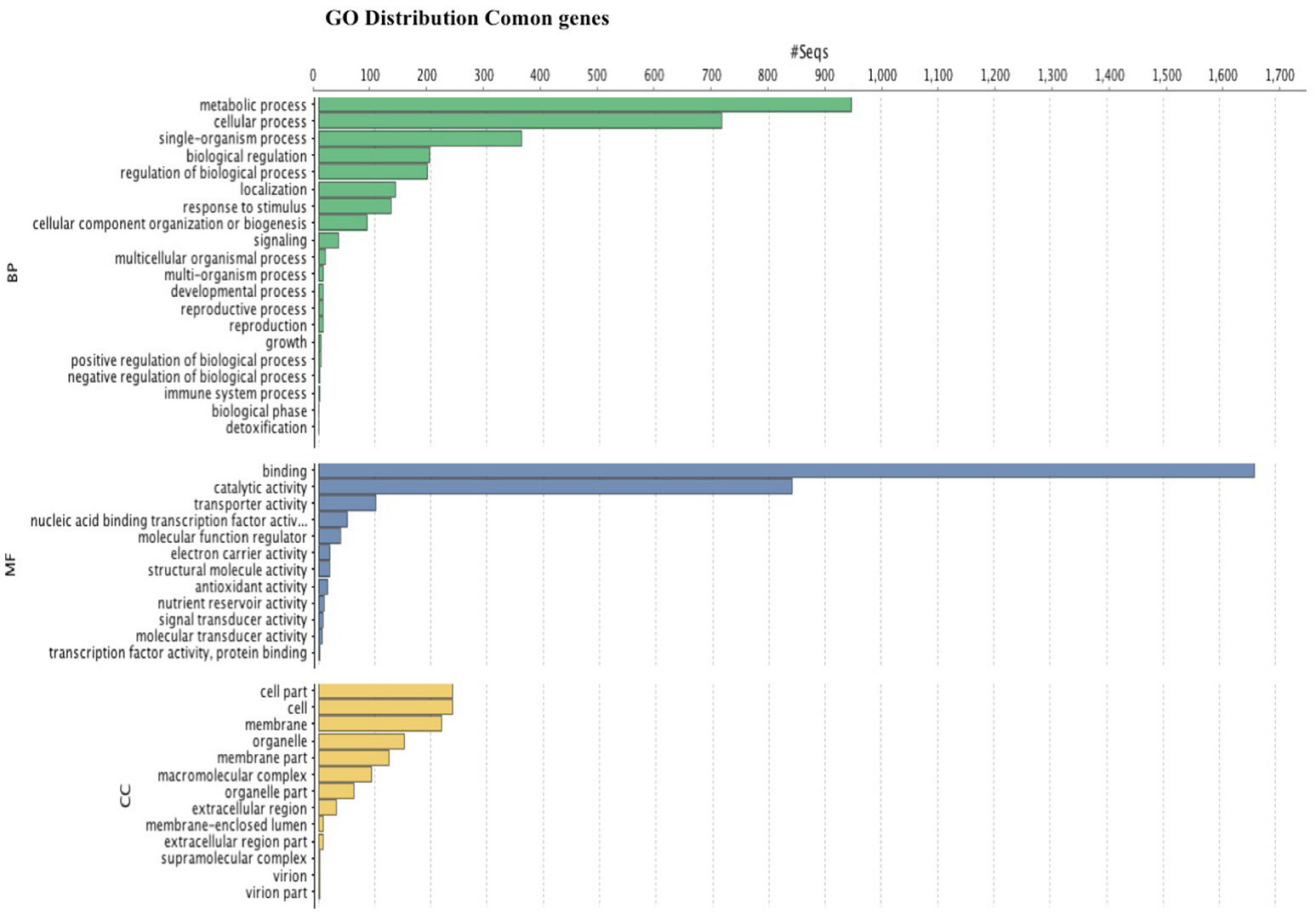
**Histogram of GO classifications of ***Rubus*** spp. unigenes**. Results are summarized for the three main categories: biological process, cellular component and molecular function.

GO analysis (Figure 6) shows that the most abundant genes are grouped in metabolic and cellular processes as well as binding, and catalytic activity. GO analysis for leaves and fruits is shown in independent figures in supplementary material.

A detailed analysis of overexpressed genes in fruits (supplementary material) indicate presence of terpenes, a very low presence of alkaloids while phenolic compounds predominate.

Overexpressed genes in leaves are related to an active photosynthesis (mostly related to photosystems I and II), and to an efficient capacity of ROS scavenging, shown in the high number of transcripts of SOD, ascorbate oxidase and glutathione reductase. Also, glutamate and glutamic acid metabolism-related transcripts are abundant in leaves as well as auxin-related genes. As regards of secondary metabolism, leaves show similar expression patterns as in fruits: alkaloid metabolism is almost absent, terpene metabolism is similar, and phenolic compounds metabolism predominate. Different to fruits, leaves show abundant transcripts of the shikimate pathway; interestingly, isoflavones, and anthocyanins-related genes are also overexpressed. Interestingly, although MATE, and ABC transporters and Glutathione-S-Transferases were expressed in leaves and fruits, only ABC and GST were overexpressed in leaves. Finally, abundant transcripts related to defense (PRs and LRRs) were also found in leaves (supplementary material).

### RT-qPCR analysis of the flavonol-anthocyanin pathway

**Figure 7:**
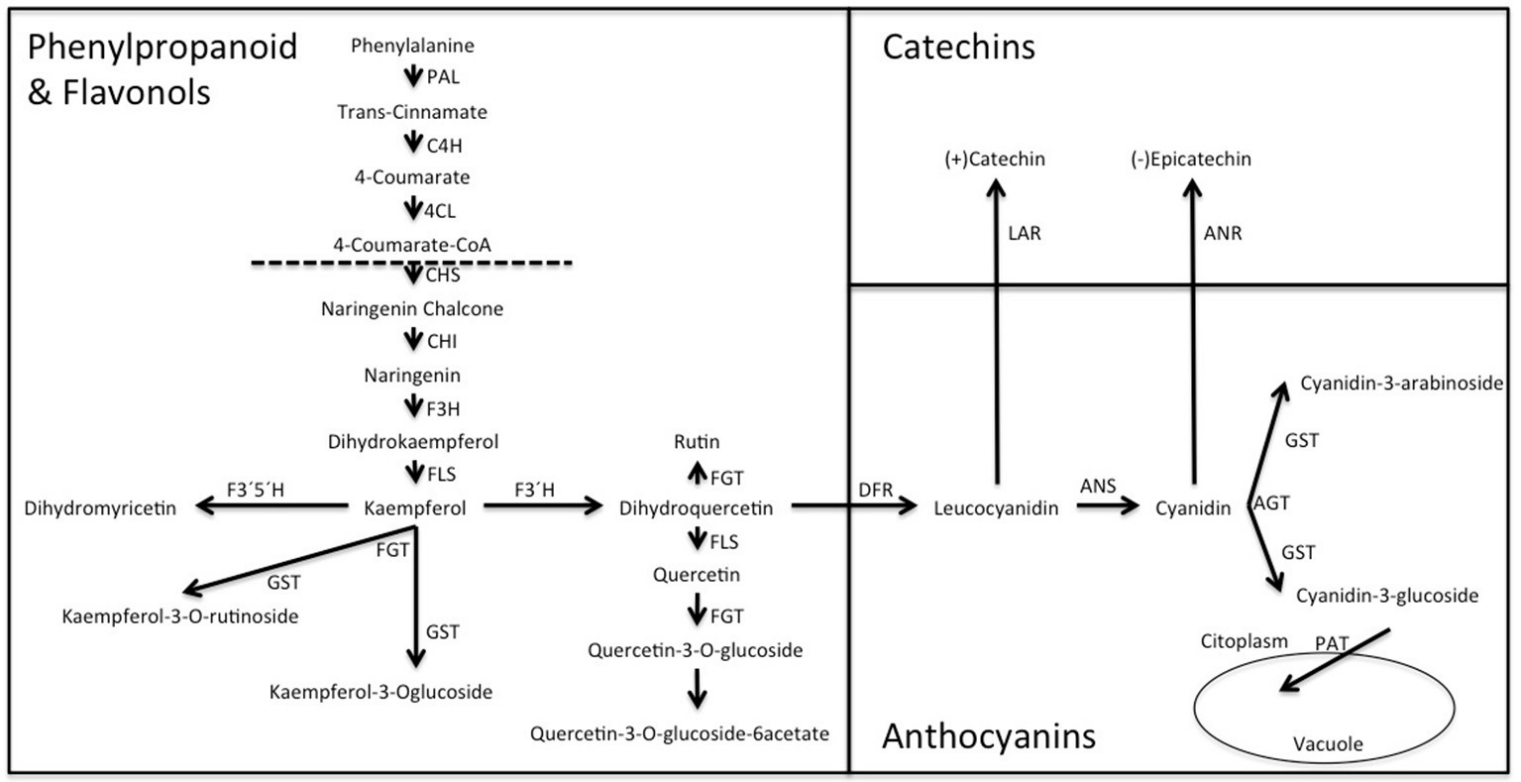
**Biosynthesis of anthocyanins, flavonols and catechins via the flavonoid pathway in ***Rubus*** spp. Var. Loch Ness**. Phenyl alanine ammonio-lyase (*RuPAL1* and *RuPAL2*), Cinammate 4 hydroxylase (*RuC4H*), 4-coumaryl: CoA ligase (*Ru4CL*), Chalcone synthase (*RuCHS*), Chalcone Isomerase1 (*RuCHI1*), Chalcone Isomerase2 (*RuCHI2*), Flavonol-3-hydroxylase (*RuF3H*), Flavonoid 3′5′hydroxylase (*RuF3*′*5'H)*, Flavonoid 3′hydroxylase (*RuF3*′*H*), Flavonol synthase (*RuFLS*), Leucoanthocyanidin reductase (*RuLAR*), Anthocyanidin reductase (*RuANR*), Dehydroflavonol reductase (*RuDFR*), Anthocyanidin synthase (*RuANS*), Glycosiltransferases (*RuGST1* and *RuGST2)*.

**Figure 8:**
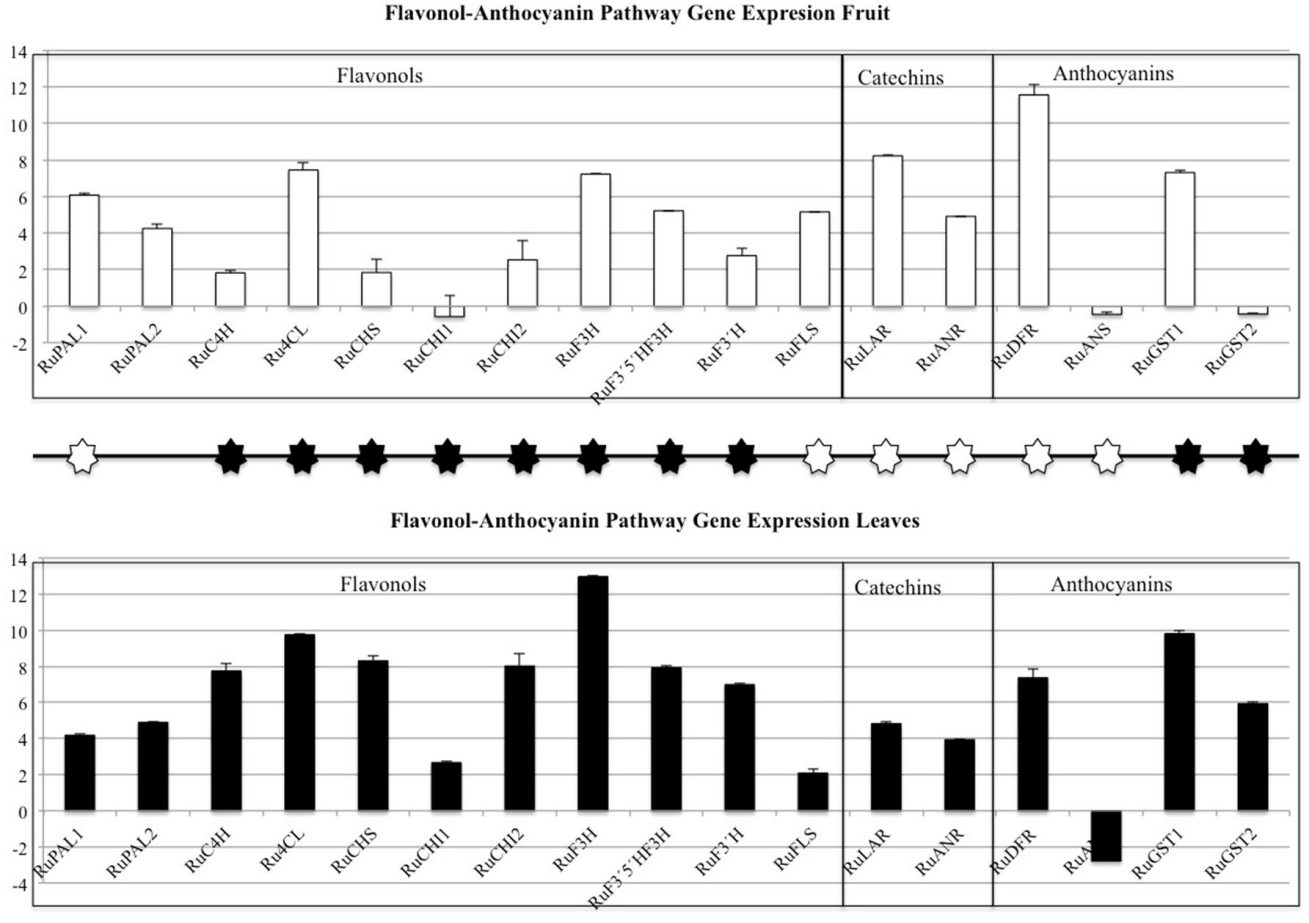
**Flavonol-Anthocyanin Pathway gene expression analyzed by RT-qPCR**. Phenyl alanine ammonio-lyase (*RuPAL1* and *RuPAL2*), Cinammate 4 hydroxylase (*RuC4H*), 4-coumaryl-CoA ligase (*Ru4CL*), Chalcone synthase (*RuCHS*), Chalcone Isomerase1 (*RuCHI1*), Chalcone Isomerase2 (*RuCHI2*), Flavonol-3-hydroxylase (*RuF3H*), Flavonoid 3′5′hydroxylase (*RuF3*′*5*′*H*), Flavonoid 3′hydroxylase (*RuF3*′*H*), Flavonol synthase (*RuFLS*), Leucoanthocyanidin reductase (*RuLAR*), Anthocyanidin reductase (*RuANR*), Dehydroflavonol reductase (*RuDFR*), Anthocyanidin synthase (*RuANS*), Glycosiltransferases (*RuGST1* and *RuGST2)*. Asterisks indicate significant differences, according to Fisher test (*p* < 0.05). Black and white asterisks indicate higher expression in leaves or fruits, respectively.

**Figure 9:**
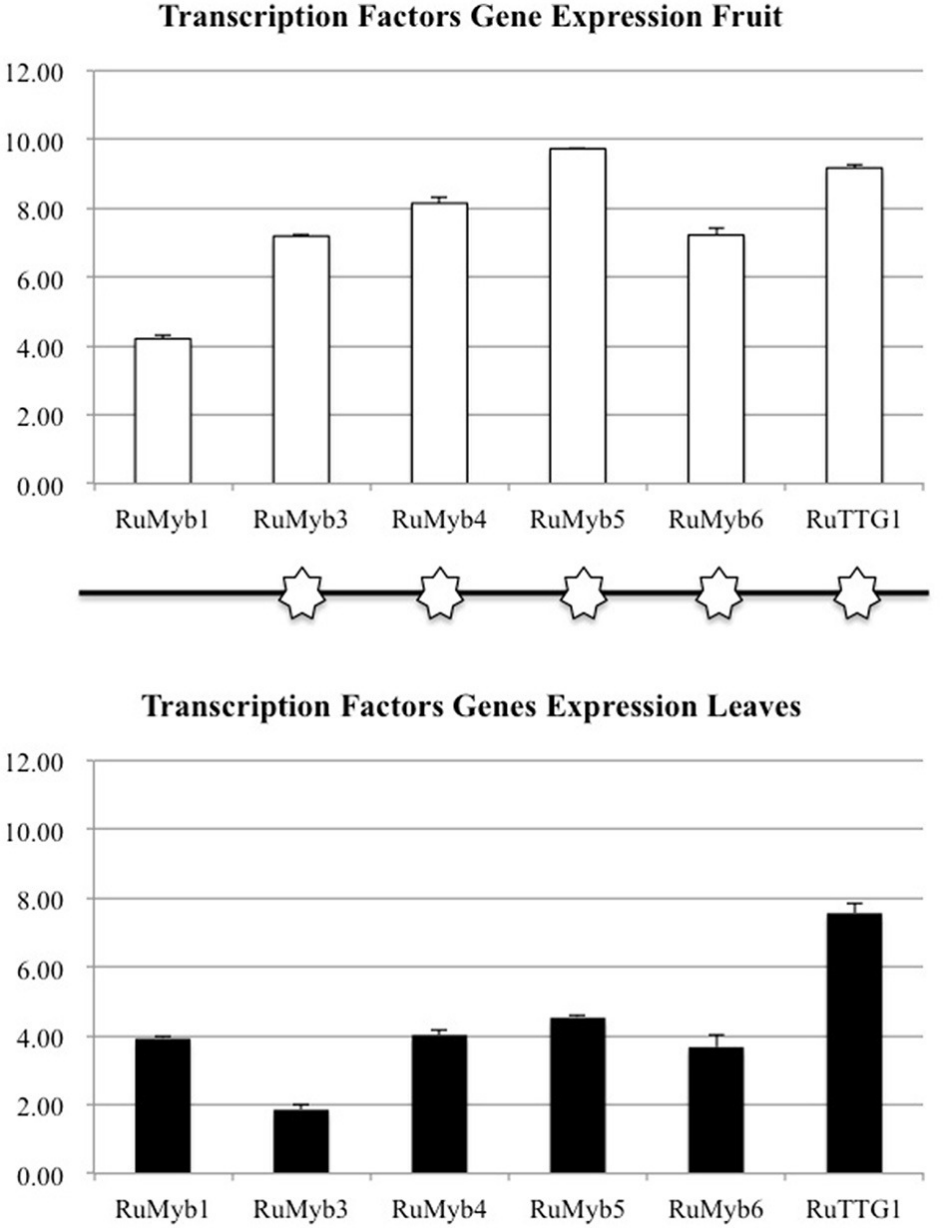
**Transcription factors gene expression flavonols anthocyanins pathway (***RuMYB1, RuMYB2, RuMYB3, RuMYB4, RuMYB5, RuMYB6, RuTTG1***, and ***RuBHLH1***)**. Asterisks indicate significant differences, according to Fisher test (*p* < 0.05). Black and white asterisks indicate higher expression in leaves or fruits, respectively.

The flavonol-anthocyanin pathway is illustrated in Figure 7. Expression of core (Figure 8) and regulatory genes (Figure 9) was studied in leaves and fruits.

As regards to core genes (Figure 8), all were expressed in leaves and fruits, except for *RuANS* that was downregulated in both. In general, expression of the flavonol-anthocyanin pathway core genes was higher in leaves. Two isoforms were studied for *RuPAL, RuCHI*, and *RuGST*. Both *RuPAL* isoforms were found in both organs, being *RuPAL1* significantly overexpressed in fruits. Expression of chalcone synthase (*RuCHS*) involved in extension of 4 hydroxy-cumaroyl-CoA side chain, showed a significant four-fold expression in leaves as compared to fruits; this expression, coupled to a significant four-fold and two-fold expression of *RuCHI2* and *RuF3H* respectively, was consistent with the higher concentration of flavonols found in leaves (Figure 8). *RuGST1* was expressed in both organs while *RuGST2* was expressed only in leaves, and these differences were significant.

Conversely to core genes, expression levels of all regulatory genes or transcription factors (TF) were higher in fruits (Figure 9). The regulatory gene's expression (Figure 9) showed that *RuMYB1, RuMYB5, and RuMYB6* had higher expression in red fruit than in leaves. The negative pathway regulator *RuMYB4*, showed high expression in red fruit while leaves showed a lower expression of this *RuMYB4*, consistent with higher expression of *RuC4H* in leaves.

### Bioactive characterization

#### Bioactives quantification

**Figure 10:**
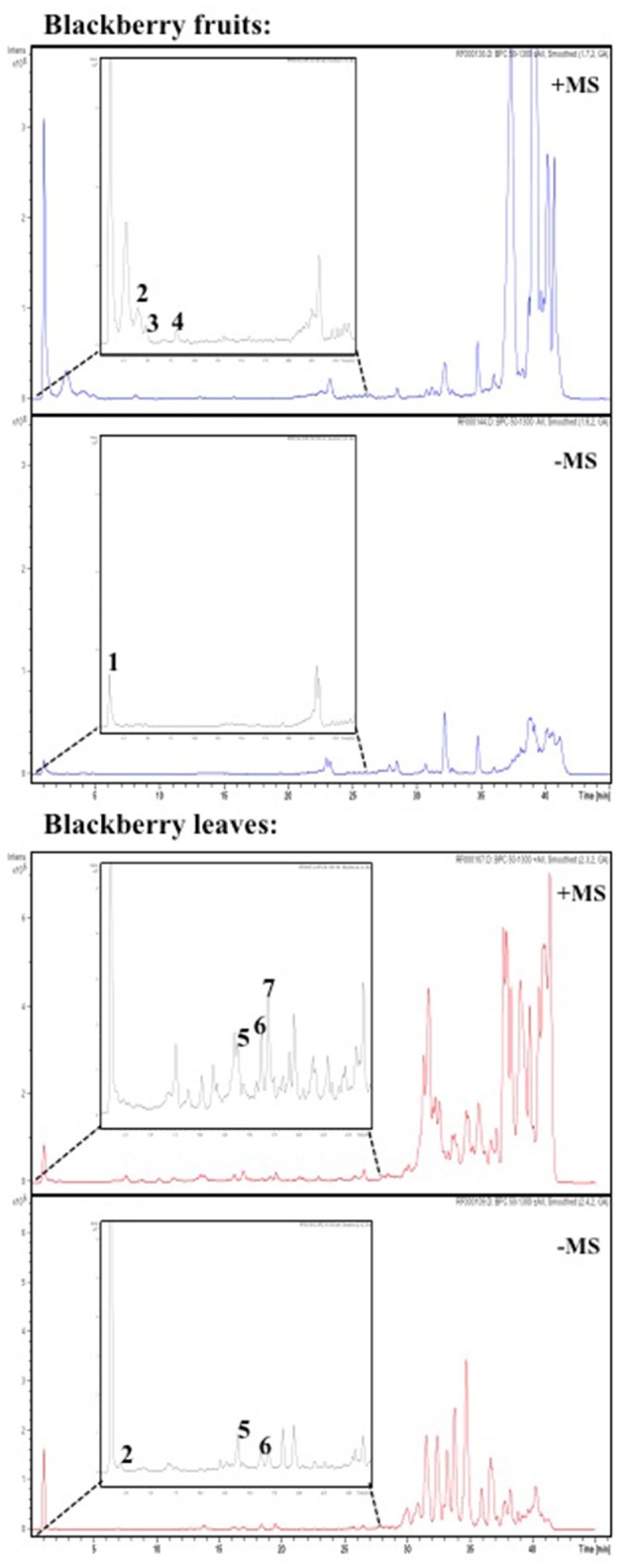
**Enlargement the HPLC chromatogram (Base Peak Chromatogram positive (+) and negative (−) ion mode) for phenolic acids and flavonoids present in blackberry fruits (A)** and leaves **(B)** samples, detected at 260 nm. Numbering of peaks refers to their identification as shown in Tables 3, 4.

Bioactives were quantified by colorimetry (Table 3) and identified by HPLC-MS (Figure 10). Red fruits contained 560 mg gallic acid equivalents (total phenols), 34 mg catechin equivalents (total flavonols), and 108 mg cyanidin-3-glucoside equivalents (total anthocyanins). Leaves showed 1,876 mg gallic acid equivalents, 296 mg catechin equivalents and 55 mg of cyanidin-3-glucoside equivalents. All results are expressed per 100 g fresh weigh. In summary, contents in leaves as compared to fruits were as follows: total phenolics were three-fold in leaves, while total flavonoids were six-fold in leaves and anthocyanins were two-fold in fruits.

**Table 3:** **Quantification of total phenolics, flavonols, and anthocyanins in blackberry fruits and leaves**.

**Sample**	**Total phenols mg gallic acid equivalents/100 g fresh weigh**	**Total flavonols mg catechin acid equivalents/100 g fresh weigh**	**Total anthocyanins mg cyanidin-3-O-glucoside acid equivalents/100 g fresh weigh**
Leaves	^*^1876.56 ± 32.47	^*^06.24 ± 6.79	28.40 ± 0.78
Fruit	560.60 ± 8.23	34.70 ± 1.41	^*^64.70 ± 1.56

*Data is the average of three replicates. Asterisks indicate significant differences, according to Fisher test (p < 0.05)*.

#### Peak identification and assignment

The LC/MS analysis was performed using defined conditions that provided a good separation profile (Figure 10). Identification and peak assignment was based on comparison of their retention time and mass spectra data with those of standards available, and the results are indicated in Tables 4, 5, where *m/z* experimental values from the BPC (Base Peak Chromatograms) obtained in negative and positive ion mode as well as the fragments in source are shown.

**Table 4:** **Mass spectral data for identification of phenolic acids, flavonols and anthocyanins in blackberry fruits (A) by LC/MS data**.

**Peak no**.	**Compound**	**Positive ionization mode (**+**)**				**Negative ionization mode (**−**)**		
		***Rt* (min)**	**Selected ion**	**m/z exp**.	Fragment ions^*^	**Selected ion**	**m/z exp**.	Fragment ions^*^
1	chlorogenic acid (3-*O*-caffeoylquinic ac.)	0.8–1.2	ND	–	–	[M-H-caffeoyl]^−^	190.6	–
2	cyanidin-3-O-glucoside	2.1–4.4	[M+H]^+^	449.0	287.0	[M-H]-	446.9	284.7
3	(+)-catechin	1.8.–4.1	ND	–	–	[M-H]-	288.8	–
7	(−)-epicatechin	4.5–5.1	ND	–	–	[M-H]-	288.8	–

* *Peaks corresponding to the aglycon moiety*.

*ND- non detected*.

**Table 5:** **Mass spectral data for identification of phenolic acids, flavonols and anthocyanins in blackberry leaves (B) by LC/MS data**.

**Peak no**.	**Compound**	***Rt* (min)**	**Positive ionization mode (**+**)**			**Negative ionization mode (**−**)**		
			**Selected ion**	**m/z exp**.	Fragment ions^*^	**Selected ion**	**m/z exp**.	Fragment ions^*^
2	cyanidin-3-O-glucoside	2.6–3.1	[M+H]^+^	449.0	287.0	[M-H]^−^	446.9	284.7
4	quercetin-3-O-rutinoside	13.6–14.1	[M+Na]^+^ [M+K]^+^	633.2 649.2	303.0 286.9 271.0	[M-H]^−^	609.0	300.7
5	kaempferol-3-O-rutinoside	15.9–16.4	[M+Na]^+^	617.2	286.9 270.9	[M-H]^−^	593.0	284.7
6	quercetin-3-O-glucoside	16.6–17.2	[M+Na]^+^ [M+K]^+^	487.1 503.0	303.0 286.9 270.9	ND	–	**–**

* *Peaks corresponding to the aglycon moiety*.

*ND- non detected*.

Due to the lack of commercial standards, most peaks have not been identified and no information about these unknown compounds has been provided. However, MS fragmentation in source has helped us to establish a tentative identification for most of the significant peaks by comparison with bibliography. Thus, caffeoyl-*O*-hexosides (*Rt* = 1.0–3.3 min) with a [M-H]^−^ ion at *m/z* 340.9 and [M+Na]^+^ and [M+K]^+^ adducts at *m/z* 365.0 and *m/z* = 381.0 respectively were found. Many caffeic acid-O-dihexoside and other caffeic acid derivatives were also found mainly in leaves (*Rt* = 20–45 min) with [M-H]^−^ ion at *m/z* 503.1, and chlorogenic acid was identified only in fruits (Table 4).

In Blackberry, characteristic flavonols were kaempferol and quercetin derivatives, and cyanidin-3-O-glucoside as the most abundant anthocyanin, and (+)-catechin and (−)-epicatechin among catechols. However, leaf and fruits showed different profiles. According to LC/MS data (Table 4) (+)-catechin, (−)-epicatechin, chlorogenic acid and cyanidin 3-O-glucoside were present in red fruits. Cyanidin-3-O-glucoside was the only compound common to leaves and fruits (Table 5). On the other hand, the flavonols quercetin-3-O-rutinoside kaempferol-3-O-rutinoside, and quercetin-3-O-glucoside were identified only in leaves. Interestingly flavonol glucosides were present only in leaves and derivatives of these flavonols, mainly quercetins, were present only in fruits.

## Discussion

Flavonoids are secondary metabolites that play a relevant role in plant defense, being of great importance against biotic (fungus, bacterias, herbivores; Koskimaki et al., 2009; Makoi et al., 2010; Kim et al., 2011) and abiotic stress (light, temperature, water supply, minerals, and CO_2_; Ramakrishna and Ravishankar, 2011). They are also beneficial compounds for human health, having different and beneficial effects as antioxidants, cardioprotective, antihypertensive, or blood glucose regulation (Lotito and Frei, 2004; Cassidy et al., 2011; Martin et al., 2011; Fuchs et al., 2016). Therefore, this study was undertaken to gain knowledge about bioactive metabolism in leaf and fruit, to understand the mechanism by which the plant synthesizes and accumulates bioactives in fruits.

Blackberries were sampled in the maximum production period, therefore, with a very active metabolism (Ramos-Solano et al., 2014); an active photosynthesis is necessary to feed carbon into growing leaves, flowers, and fruits. Consistent with this statement, abundant transcripts related to photosynthesis were found in leaves, among which are representatives of ferredoxin, which intervenes in the cyclic and acyclic photophosphorylation, as well as transcripts responsible for the formation of proteins of photosystems I and II, and photosynthetic pigments, mainly chlorophylls A and B.

This active photosynthesis supports an active glutamate and glutamic acid metabolism. Related transcripts were abundant in leaves supporting high activity on N assimilation (Vance et al., 1995), and consistent with plant growth and productivity. The auxin-related genes also support an intense growth activity in the vegetative organs (Went, 1933).

Interestingly, defensive metabolism was also very active, and endochitinases (*RuPR4*), endoglucanases (*RuPR2*), endoglucosidases, (*RuPR1*), subtilisin, proteases, and thaumatine genes are noticeably expressed, along with other minoritary antimicrobial compounds. This active defensive metabolism is consistent with the powdery mildew outbreak in production greenhouses reported by Agricola El Bosque at that time, and reflects defensive metabolism that enables plant adaptation to environmental conditions.

As regards of secondary metabolism, leaves show similar expression patterns as in fruits: very low alkaloid metabolism, similar terpene metabolism, with a predominance of phenolic compounds metabolism. As secondary metabolites are highly apolar, they usually appear as sugar-bound heterosides, calling for high presence of glycosylating enzymes as well as a high activity related to vacuolar transport (Figures 4, 5), where they are stored (Yauk et al., 2014) as highlighted by Interproscan data (Figures 4, 5).

According to transcript abundance, terpenes are present mainly as monoterpenes (eugenol and geraniol) which have been reported to play a role in plant defense in leaves in other plant species as Picea (Faldt et al., 2003), pine (Himejima et al., 1992), or pistacia (Monaco et al., 1982) and in flavor in fruits as honeysuckle (Chmiel et al., 2013) or strawberry (Araguez et al., 2013). Also the terpene derived plant growth regulators abscisic acid and gibberellins are abundant, consistent with the physiological status of the plant in the flowering state.

An insight into phenolic compounds reveals that phenylpropanoids, flavonoids and anthocyanins biosynthetic genes are the most abundant. Considering the abundance of transcripts of the shikimate pathway, auxin biosynthesis and flavonol-anthocyanin pathway in leaves, data suggests that part of the metabolic flux is directed to feed auxin synthesis at the chorismic acid branching point, in addition to feeding flavonol-anthocyanin synthesis (Jorgensen et al., 2005).

In a previous work a ripening study was performed, showing changes in flavonol-anthocyanin pathway core genes expression, and bioactive accumulation in the three stages (green, red, black) of maturation in fruit (Garcia-Seco et al., 2015). In this work, fruit flavonoid, and anthocyanin biosynthesis was demonstrated. However, the high levels of catechins in green fruit without an increase in the expression of the corresponding biosynthetic genes suggested that catechins were probably originated in another part of the plant; this hypothesis was further supported in the other maturation stages in which, the accumulation of bioactives is not fully supported by the increase in gene expression. Finally, changes in gene expression along maturation were more evident in the red stage. Based on these facts, the present experiment was carried out to explore the possibility of flavonols translocation from leaves to growing fruits. The working hypothesis was set to demonstrate that leaves would actively synthetize flavonols and these would be translocated to supply anthocyanin synthesis in fruits, so leaf samples were taken in the same moment, when plants showed green, red and black fruits. To reinforce our hypothesis, leaf bioactives were also quantified at early flowering, when no fruits were present, resulting in lower levels of total phenolics and total flavonoids (data not shown). This data supports our hypothesis of increased leaf flavonol synthesis along the plant's cycle (Wang et al., 2009) in order to supply the fruit in its early stages.

Our results show that flavonol biosynthesis takes place in different organs, therefore they need to be accumulated and/or transported. An overexpression of chalcone synthase (*RuCHS*) coupled to *RuCHI2* and *RuF3H*, is consistent with the higher concentration of flavonols found in leaves (Table 3) and supports the active synthesis therein. Interestingly, fruits and leaves showed expression of the MATE, ABC transporters, glutathione-S-transferases and glycosyltransferases (GSTs) involved in intracellular transport and glycosylation of anthocyanins, proanthocyanidins, and flavonols to vacuoles (Gomez et al., 2011; Zhao, 2015; Perez-Diaz et al., 2016). Therefore, flavonols and catechins are being actively glycosylated and consequently stored in each organ. However, the higher concentration of flavonols in leaves together with transporters (ABC and glutathione-S-transferases) overexpression and the *RuGST1* and *RuGST2* qPCR expression (Figure 8), indicates that they may as well be translocated to other tissues, consistent with the reported long distance transport demonstrated in *A. thaliana* by MATE and ABC transporters (Buer et al., 2007). Consequently, based in data from InterProScan Families Distribution overexpressed in leaves (Figure 4), the abundance of UDP-glycosiltransferases and the overexpression of ABC and glutathione-S-transferases transporters in leaves reinforce the hypothesis that flavonols synthetized in leaves are being translocated to fruits (Petrussa et al., 2013).

The overexpression of the isoform *RuPAL1* over the *RuPAL2* in fruits (Figure 8) is consistent with the complex role of this enzyme in directing the carbon flux to the flavonol pathway in key points of fruit ripening (Garcia-Seco et al., 2015; Dastmalchi et al., 2017) and further supports the initial activation of the flavonol synthesis in fruits, together with the presence of cholrogenic acid identified therein (Table 4). Interestingly, the almost two-fold expression of *RuDFR* and *RuLAR* in fruits suggests that accumulation of catechins (Table 3) is taking place in fruits, as these compounds are usually accumulated in seeds (Zhang et al., 2014) and is supported by the presence of catechins only in fruits (Tables 4, 5), probably synthetized from the quercetins originated in leaves. The downregulation of *RuANS* is consistent with the mid ripe status of blackberries, that still have not started to massively accumulate anthocyanins (Garcia-Seco et al., 2015).

Different branches of the flavonoid pathway are highly regulated at the transcriptional level, especially by MYB transcription factors (Czemmel et al., 2012). Transcription factors play an important role in flavonoid biosynthesis (Matsui et al., 2007) regulating some genes co-expression to stimulate or inhibit the accumulation of flavonoids. Based on a previous study on blackberry (Garcia-Seco et al., 2015), homologous regulatory genes (*RuMYB1, RuMYB3, RuMYB4, RuMYB5, RuMYB6, RuTT2*) were found and their expression was also evaluated (Figure 9).

The higher expression of the activating flavonol biosynthetic pathway transcription factor genes in fruit as compared to leaves (Figure 9; *RuMYB*1, *RuMYB*3, and *RuMYB*5) is consistent with the massive synthesis of anthocyanin precursors in fruits that is eventually going to take place during ripening. *RuMYB1, RuMYB3*, and *RuMYB5* are positive regulators of *RuDFR, RuANR*, and *RuLAR*, leading to increased catechin synthesis, precursors of proanthocyanidins, that are accumulating in fruits (Table 4). *RuMYB1* encodes a homolog of *FaMYB10*, which plays a major role in the regulation of anthocyanin and phenylpropanoid metabolism during ripening of *Fragaria x ananassa* fruits (Kadomura-Ishikawa et al., 2015). *RuMYB5* is homologous to *AtMYB5* in *Arabidopsis*, and the *AtMYB5* protein interacts with *TTG1* and *TT2*, two important transcription factors regulating the proanthocyanidin biosynthetic pathway (Baudry et al., 2004). The expression of the *RuMYB5* in leaves is low, while it is quite high in fruits, consistent with the better activation of core pathway for proanthocyanidin synthesis in fruits (*RuDFR, RuANR, RuLAR*; Figure 8). Therefore, the enhanced expression of *RuMYB1, 3*, and *5* on fruits reflects an activation of the pathway leading to proanthocyanidins, data supported by the HPLC-MS analysis, where chlorogenic acid, cyanidin-3-O-glucoside and the catechols (+)-catechin and (−)-epicatechin, precursors of proanthocyanidins were identified in fruits (Tables 4, 5).

*RuMYB6* encodes a homolog of *GmMYB12B2* from *Glycine max*, a transcription factor that activates *CHS* in soybean and *Arabidopsis*. In fact, *GmMYB12B2*, induces expression of *PAL, CHS*, and *FLS* in *Arabidopsis* (Li et al., 2013). Our results show the activation of *RuPAL* and *RuFLS* expression in fruits. The higher expression of *RuMYB6* in red fruit as compared to leaves, suggests that the massive synthesis of anthocyanins associated to maturation is going to start, as anticipated by the higher expression of *RuMYB6* in fruits; until this happens, flavonoids are synthesized in leaves and then translocated to fruits.

*RuMYB4* encodes a homolog of *AtMYB4*, an inhibitor of *C4H* expression in *Arabidopsis* (Jin et al., 2000; Zhao et al., 2007). It shows high expression in red fruit consistent with the repression of *RuC4H* in fruits, while leaves show a lower expression of this *RuMYB4*, consistent with higher expression of *RuC4H* therein, and further supports the hypothesis.

In summary, transcription factors expression in fruits is higher than in leaves suggesting that the onset of maturation has been activated and will therefore need to receive an active precursor supply for anthocyanin synthesis and accumulation. The low expression of TF in leaves suggests that the pathway has already been activated, and therefore, TF have already played their role, so expression is lower; however, activity of core genes is reflected on flavonoid synthesis to fulfill fruit demand, and transport is supported by the overexpression of ABC and glutathione transferases genes in leaves and enhanced expression of *RuGST*s in leaves. Finally, the six-fold concentration of flavonols in leaves as compared to fruits, and the accumulation of flavonols only in leaves as compared to catechins and cyanidin-3-glucoside, their downstream biosynthetic products in fruits, strongly support the translocation hypothesis to supply flavonol scaffoldings for first steps of fruit biosynthesis, which will be strongly activated when fruits achieve the mature state.

In conclusion, data of gene expression presented above is consistent with flavonol accumulation in red fruits that will be transformed into anthocyanins when fully mature (black; Garcia-Seco et al., 2015); conversely, in leaves the last part of the pathway (responsible of the production of flavonols and anthocyanins), is more expressed which makes sense, as these compounds also participate in plant defense. Moreover, these values are consistent with the expression levels of core and regulatory genes, supporting the working hypothesis of a strong synthesis of flavonols in leaves that are translocated to fruits to supply flavonol scaffoldings for first steps of fruit biosynthesis which synthesis will be strongly activated upon ripening; if this was so, flavonols will be translocated from leaves to fruits, creating a really efficient system for anthocyanin accumulation.

## Author contributions

Conception and design of the work: FG and BR. Field work and sampling: JL, BR, EG. Analysis of the samples: AGV, AG, EG. Interpretation of the data: AGV, BR, JL, AG, EG. Drafting the work and revising: BR. Final approval of the version to be published: BR, FG.

## Funding

Proyect funded By Ministerio de Economía y Competitividad: AGL-2013-45189-R. Grant reference: BES-2014-069990.

### Conflict of interest statement

The authors declare that the research was conducted in the absence of any commercial or financial relationships that could be construed as a potential conflict of interest.
