# Supplementary figures and images for: Transcriptomics, Targeted Metabolomics and Gene Expression of Blackberry Leaves and Fruits Indicate Flavonoid Metabolic Flux from Leaf to Red Fruit
Source: Front Plant Sci. 2017 Apr 6;8:472. doi: 10.3389/fpls.2017.00472 (PMC5382209; doi:10.3389/fpls.2017.00472)

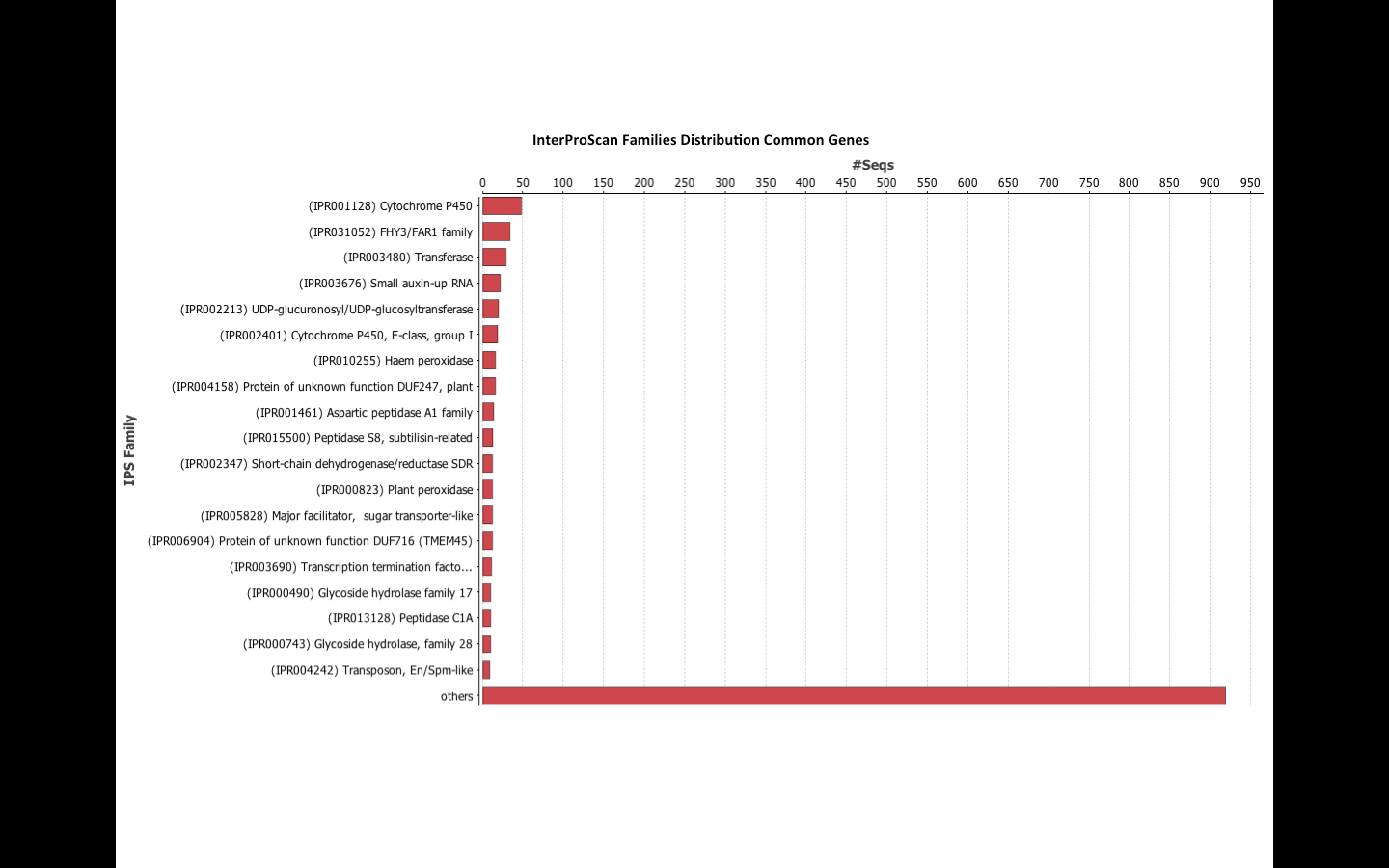

Supplement: Supplementary file 4 [file Image1.JPEG]

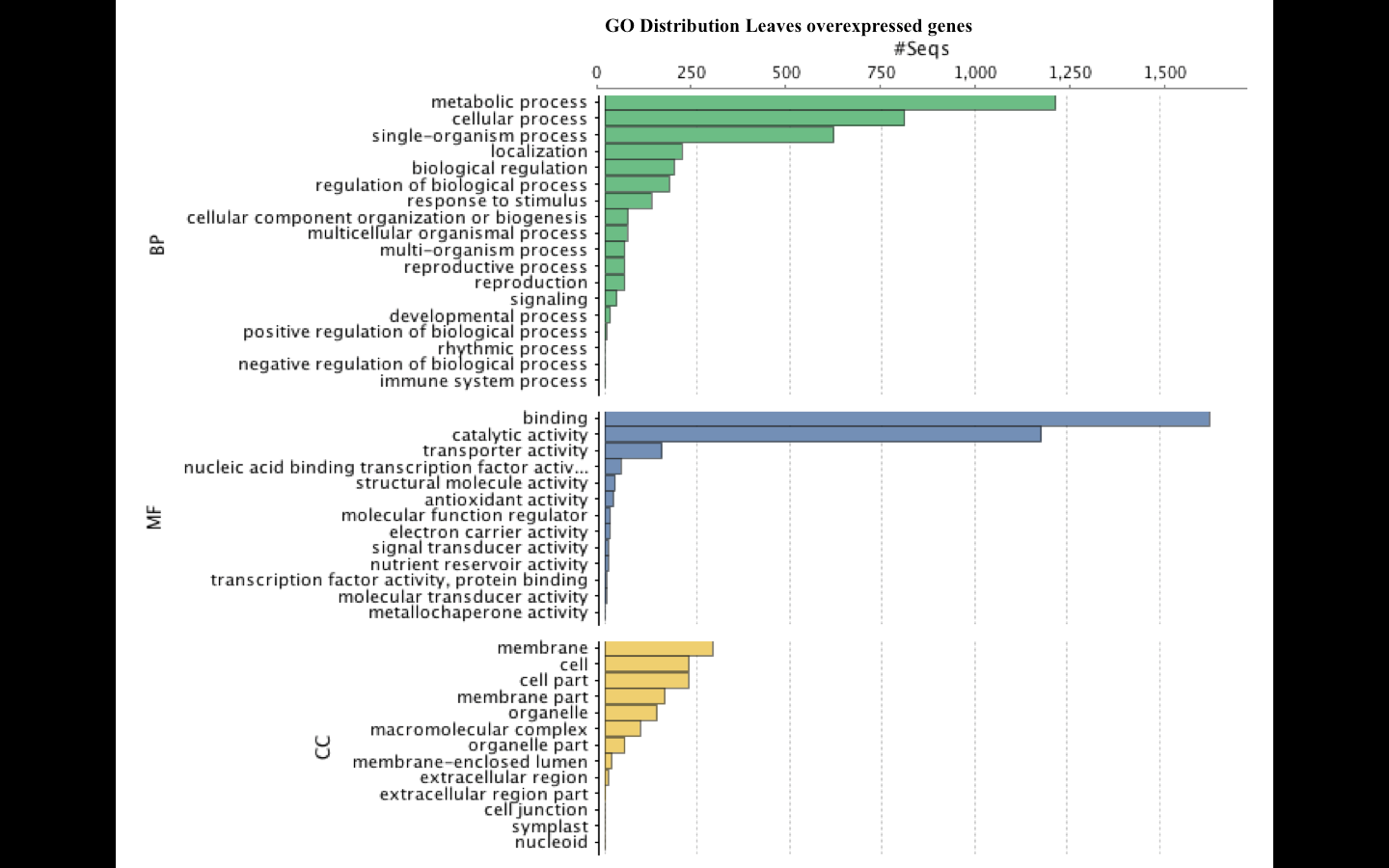

Supplement: Supplementary file 5 [file Image2.JPEG]

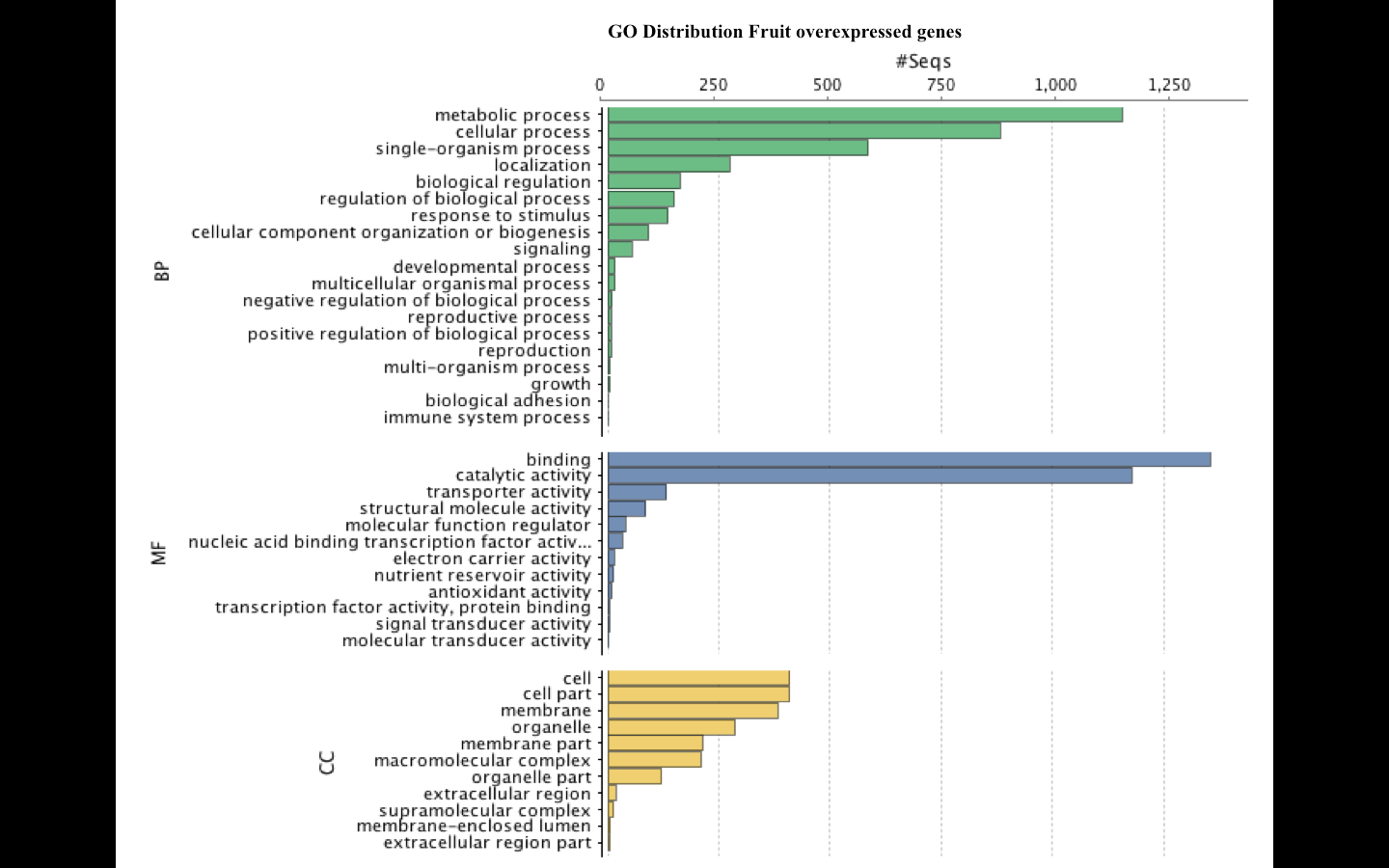

Supplement: Supplementary file 6 [file Image3.JPEG]
